# Defense Responses to Short-term Hypoxia and Seawater Acidification in the Thick Shell Mussel *Mytilus coruscus*

**DOI:** 10.3389/fphys.2017.00145

**Published:** 2017-03-09

**Authors:** Yanming Sui, Yimeng Liu, Xin Zhao, Sam Dupont, Menghong Hu, Fangli Wu, Xizhi Huang, Jiale Li, Weiqun Lu, Youji Wang

**Affiliations:** ^1^Department of Biology, College of Fisheries and Life Science, Shanghai Ocean UniversityShanghai, China; ^2^Key Laboratory of East China Sea and Oceanic Fishery Resources Exploitation, Ministry of Agriculture of China, East China Sea Fisheries Research Institute, Chinese Academy of Fisheries SciencesShanghai, China; ^3^Key Laboratory of Exploration and Utilization of Aquatic Genetic Resources, Ministry of EducationShanghai, China; ^4^Department of Biological and Environmental Sciences, Sven Lovén Centre for Marine Sciences, University of GothenburgFiskebäckskil, Sweden

**Keywords:** pH, hypoxia, byssus, mussel, defense response

## Abstract

The rising anthropogenic atmospheric CO_2_ results in the reduction of seawater pH, namely ocean acidification (OA). In East China Sea, the largest coastal hypoxic zone was observed in the world. This region is also strongly impacted by ocean acidification as receiving much nutrient from Changjiang and Qiantangjiang, and organisms can experience great short-term natural variability of DO and pH in this area. In order to evaluate the defense responses of marine mussels under this scenario, the thick shell mussel *Mytilus coruscus* were exposed to three pH/pCO_2_ levels (7.3/2800 μatm, 7.7/1020 μatm, 8.1/376 μatm) at two dissolved oxygen concentrations (DO, 2.0, 6.0 mg L^−1^) for 72 h. Results showed that byssus thread parameters, such as the number, diameter, attachment strength and plaque area were reduced by low DO, and shell-closing strength was significantly weaker under both hypoxia and low pH conditions. Expression patterns of genes related to mussel byssus protein (MBP) were affected by hypoxia. Generally, hypoxia reduced MBP1 and MBP7 expressions, but increased MBP13 expression. In conclusion, both hypoxia and low pH induced negative effects on mussel defense responses, with hypoxia being the main driver of change. In addition, significant interactive effects between pH and DO were observed on shell-closing strength. Therefore, the adverse effects induced by hypoxia on the defense of mussels may be aggravated by low pH in the natural environments.

## Introduction

Anthropogenic CO_2_ emissions are driving increases in the net CO_2_ uptake by the oceans. Consequently, current average oceanic pH values are already 0.1 unit lower than pre-industrial values and are expected to decrease an extra 0.3–0.4 unit by the end of 2,100 (Feely and Millero, 2004; Orr et al., 2005). Decreased pH results in profound modification of the seawater carbonate chemistry including a reduction of the calcite saturation state [Ω_cal_ = ([Ca^2+^][${\text{CO}}_{3}^{2-}$]/K_sp_), where sp is the solubility product at a assumed pressure, temperature and salinity]. This phenomenon is named “ocean acidification” (OA). At the same time, increasing global temperatures (global warming), closely related to changes in atmospheric CO_2_ concentrations, also lead to a more stratified surface ocean, cutting down exchange between deep and surface seawaters, resulting in expansion of oxygen-limited zones. Compared with colder waters, DO levels in warmer waters are lower, and global warming enhances the stratification of the upper ocean. These two factors decrease the supply of oxygen to the deeper parts of the ocean, spreading out hypoxic zones (Pörtner et al., 2005; Keeling et al., 2009; Feely et al., 2010; Stramma et al., 2010). Moreover, eutrophication usually exacerbates hypoxia in offshore waters (Levin et al., 2009).

In East China Sea, a >12,000 km^2^ hypoxic zone was reported, comparable to the largest coastal hypoxic zones observed in the world (Chen et al., 2007). Such region is predicted to be also strongly impacted by ocean acidification (Cai et al., 2011; Chou et al., 2013; Melzner et al., 2013). Within this area, the Shengsi island is famous for its largest mussel aquaculture in China. This area is located at the river mouth of Changjiang and Qiantangjiang, and receives much nutrient, resulting in eutrophication and biological CO_2_ production, especially in wet season. Consequently, mussels can experience great natural variability of DO and pH in short-term (Chen et al., 2007; Li et al., 2014). Recently mass mussel mortalities frequently occurred in summer possibly due to short-term hypoxia or large pH fluctuation, for example, DO < 3 mg L^−1^ and low pH 7.3–7.7 lasting for 3–7 days were found in our sampling area, the Shengsi island.

A growing number of studies has investigated the biological impact of hypoxia and ocean acidification, separately. OA has the potential to negatively impact marine organism (Melzner et al., 2009). For example, OA can decrease calcification rate (e.g., Kottmeier et al., 2016) and induce oxidative stress (e.g., Moolten, 2009). In the Akoya pearl oyster *Pinctada fucata*, calcification was proved to decrease due to OA (Liu et al., 2012). In addition, CO_2_-driven seawater acidification has been demonstrated to affect internal bicarbonate homeostasis, acid-base regulation as well as energy metabolism in many marine animals (Melzner et al., 2009; Hu et al., 2011, 2013, 2014a,b, 2016a,b; Stumpp et al., 2012a,b). The thick shell mussel *Mytilus coruscus* has been shown to produce more ROS under OA (Sui et al., 2016b). However, OA did not severely affect *Mytilus galloprovincialis* although reduced growth rates, lower acid-base regulation capacities and damage of the periostracum cover were found especially in summer when mussels are exposed to elevated temperature (Gazeau et al., 2014). Minor impacts of OA on growth and calcification were also observed in *M. edulis*, whereas food availability played a more important role in its tolerance to OA (Thomsen et al., 2013). In some areas of the Western Baltic Sea, seawater pCO_2_ can be high for prolonged periods due to upwelling of CO_2_ rich waters. Thomsen et al. (2010) showed that the blue mussel *M. edulis* from Kiel Fjord can maintain normal rates of somatic and shell growth at high pCO_2_ (1,400 μatm), indicating a potential pre-adaptation of *Mytilus* species to pH fluctuations. Low DO can occur naturally in estuaries, and the impacts of hypoxia on marine organism are also widely investigated (Keeling et al., 2009). Physiological responses to hypoxia are species specific (Pörtner et al., 2005; Seibel et al., 2014). For example, some bivalve species living in intertidal zone are tolerant to hypoxia because they are adaptive to variable habitats, whereas in other species (i.e., the target species *Mytilus coruscus* inhabits in subtidal zone) that are not regularly exposed to air, hypoxia can strongly impact their physiology, behaviors and distributions (Zhang et al., 2010; Wang et al., 2012). For example, hypoxia-inducible factors a (HIFa) and Hif-prolyl hydroxylases (PHD) proteins in the marine mussel *Mytilus galloprovincialis* were modulated by oxygen availability in a time-dependent manner with trends comparable to mRNA expression patterns (Giannetto et al., 2015).

A limited number of studies published to date have focused on the response to combined seawater hypoxia and acidification (Melzner et al., 2013; Gobler et al., 2014; Hernroth et al., 2015; Jakubowska and Normant, 2015; Sui et al., 2015). However, it is essential to understand the impacts of multiple stressors because these environmental changes do not change in isolation and their effects can be additive, synergistic or antagonistic (Breitburg et al., 2015). Gobler and Baumann (2016) reviewed the combined effects of pH and DO on the biological responses (e.g., survival, growth, metabolism) in numerous marine species. Additive negative effects seem to be the most common, but synergistic negative effects were also reported. To date, the interaction between pH and DO is poorly understood, and needs to be clarified.

*Mytilus coruscus* is a widely distributed mussel in coastal waters of the Yellow Sea and the East China Sea, and cultured as an important economic shellfish species, especially around the Shengsi island (Liao et al., 2013). This calcifier inhabits coastal ecosystem where it attaches to hard substrates in subtidal zones and forms large subtidal beds structuring the whole macrofauna communities. They attach to rocks under the water by byssal threads, preventing mussels from being removed and swept away by predators or waves. Additionally, the adductor muscle plays important behavioral functions such as opening and closing the shell, as well as being an energy reserve (Aoki et al., 2010). In general, healthy mussels close their valves tightly in response to various stimuli (e.g., wave and predator). Therefore, the strength of the adductor muscle and the byssus secretion ability in mussels reflect their defense level. In previous studies, we have investigated the energy budget and haemocyte responses of *M. coruscus* under simultaneous seawater acidification and hypoxia. We have shown that both stressors can negatively impact their physiology and immune response (Sui et al., 2016a,b). However, the mechanism and other aspects of the defense responses of mussels exposed to combined low pH and DO are still unclear.

In the present study, we evaluated strength of the adductor muscle and byssus performance of *M. coruscus* exposed to three pH values at two DO concentrations. The byssus consists of a thread, a flared adhesive plaque, and the plaque tip at the end of each thread that attaches to foreign surface. Byssus contains high concentrations of protein secreted by foot glands, among of which are mussel byssus proteins (MBP) 1, 5, 7, 13. MBP1 is only found in the plaque, MBP7 is only found in the thread, and both MBP5 and MBP13 are found in both the plaque and the thread (Sun et al., 2014). They are playing key roles in forming byssus. Hereby, gene expression patterns of mussel byssus proteins were also studied to better understand the defense mechanisms of *M. coruscus* exposed to low pH and DO.

## Materials and methods

### Animal collection

Wild thick shell mussel *M. coruscus* (50 ± 6 mm shell length, 125.0 ± 15.5 mg dry tissue weight) were collected from a mussel raft at the Shengsi island in Zhejiang Province (30° 33′ 00.945″ N, 121° 49′ 59.757″ E), China. The handling of mussels was conducted according to the regulations of the animal welfare for scientific research made by the Institutional Animal Care and Use Committee (IACUC) of Shanghai Ocean University. These mussels were naturally growing in a fish farm and no specific permissions were required for collection or conducting our experiments. Mussels presenting no shell damage were selected and barnacles on the shells were gently removed. Mussels were first acclimated to laboratory conditions, mimicking the Shengsi island environment at the sampling time: temperature 20°C, salinity 25.0, pH 8.1 and DO 6 mg O_2_ L^−1^ for 2 weeks in open-flow tanks (500 L). They were fed twice daily with the microalgae *Chlorella spp* (algal strain from Shanghai Ocean University, 25,000 cells mL^−1^, ca. 3% of the mussel dry weight).

### Experimental design and seawater chemistry

DO and pH were tested in a fully crossed design: two DO levels (2 mg L^−1^ as hypoxic condition and 6 mg L^−1^ as normoxic condition); three pH levels (pH 8.1 as the present average pH, pH 7.7 as the predicted average pH by the end of this century (IPCC, 2007) and extreme low value of the present natural variability in the sampling area (Li et al., 2014), and pH 7.3 as extreme low pH relevant for hypoxic zones by the year 2,100 (Cai et al., 2011). The experiment lasted for 3 days in triplicates for each treatment. In each replicate, 30 mussels were transferred to 30 perforated plastic containers (100 mL), and immerged into a 30 L tank.

DO was manipulated by injections of either N_2_ or air directly into the water via an O_2_ regulator (Loligo Systems Aps, Tjele, Denmark). The aquarium was sealed to prevent external disturbance. The O_2_ controller automatically allowed to maintain a stable O_2_ and DO level in each tank. The low pH was achieved by addition of pure CO_2_ using pCO_2_/pH feedback STAT systems (DAQ-M) connected to pH meters (WTW pH 3310) equipped with pH electrodes (SenTix 41) and operated by CapCTRL software (Loligo Systems Inc.). Salinity was regularly measured using a refractometer (S/Mill-E, Atago, Itabashi-Ku). Total alkalinity (A_T_) was measured by titration. Seawater carbonate chemistry parameters, including dissolved inorganic carbon (DIC), pCO_2_, saturation states of calcite (Ω_ca_) and aragonite (Ω_ar_) were calculated from A_T_ and pH_NBS_ using CO_2_sys (Robbins et al., 2010) using K1 and K2 from Mehrbach et al. (1973) and refitted according to Millero (2010).

### Sample collection for RNA extraction and cDNA synthesis

To examine the expression of mussel byssus protein (MBP) genes during exposure to hypoxia and acidification, the foot gland was collected at 0, 2, 4, 8, 12, 24, 48, and 72 h. At each sampling time point, three mussels were randomly sampled from each replicate.

Total RNA was isolated from the foot gland using Trizol (Invitrogen, USA) following the manufacturer's protocol (Sun et al., 2014). The purity of the extracted RNA was determined by the OD260 nm/OD280 nm ratio, with expected values between 1.8 and 2.0. All of the RNA samples were treated with RNase free-DNase I (Takara, Japan) to remove residual genomic DNA before being reverse transcribed into cDNA using random hexamer primers and MMLV Reverse Transcriptase (Takara, Japan) according to the manufacturer's instructions.

### Quantitative real-time PCR analysis

The quantitative real-time PCR (qRT-PCR) was carried out in 96-well qPCR plates by an ABI PRISM 7500 detector (Applied Biosystems, Foster City, CA). Specific primers for MBP1, MBP5, MBP7, MBP13 and β-actin transcripts were designed using the mussel *M. coruscus* foot gland mRNA sequences deposited in GenBank: MBP1 (GO898529.1), MBP5 (GR217715.1), MBP7 (GR277545.1), MBP13 (GE759737.1) and β-actin and Primer Premier 5.0. Primers used in this study were synthesized commercially (Sangon Biotech, Shanghai, China) (Table 1) and optimization and validation of primers for qRT-PCR were tested according to standard ABI protocols. qRT-PCR was carried out using NovoStart® SYBR qPCR SuperMix (NovoStart, Shanghai, China) according to manufacturer's instructions. For each sample, three reactions were conducted along with no-template controls. qRT-PCR parameters were 95°C, for 30 s followed by 40 cycles at 95°C, for 5 s, and 60°C, for 34 s. The gene expression levels were calculated using the comparative threshold cycle (Ct) method and expressed as 2^−ΔΔCt^ normalized to the β-actin gene expression (Liao et al., 2013; Liu et al., 2014, 2015).

**Table 1:** **Primers sequences of genes used in real-time PCR analysis**.

**Gene**	**Primer sequence (5′-3′)**	**GenBank accession number**
MBP-1-F	CCGTGTCAGTGTATCTCAACTC	GO898529.1
MBP-1-R	CTCGGCAAGTAAGTCTGCTTAT	
MBP-5-F	GCATTTCACGGAGGAAGTAGAT	GR217715.1
MBP-5-R	CCCGAATACGCTACACCATAAG	
MBP-7-F	TCTGGCCCATCGTTCATATTAC	GR277545.1
MBP-7-R	CACTCGCTGTGCAACTCTAT	
MBP-13-F	ATGTTGGAGATCTTGGGAATGT	GE759737.1
MBP-13-R	CAGGATTGACCTGTCTGATGTT	
β-actin-F	ATGAAACCACCTACAACAGT	GO898671.1
β-actin-R	TAGACCCACCAATCCAGACG	

### Measurement of byssus performance

Byssus performance was assessed using methods adapted from a previous study (Wang et al., 2012). Three mussels from each tank were randomly selected after 72 h, and four parameters were measured: (i) the number of byssus thread/byssus plaque; (ii) thread length using Vernier caliper (precision of 0.1 mm); (iii) byssus thread diameter measured 1–1.5 mm from the adhesive disk using a dissecting microscope equipped with an ocular micrometer (Nikon, precision of 0.01 mm); and (iv) byssus plaque area examined under a light microscope and measured using an image analyzing software (Image J 1.43 u).

### Measurement of byssus attachment strength

Following a 72 h exposure to the different treatments, three mussels per tank were sampled and byssus strength was measured. Prior to measurement, the perforated plastic containers attached by byssus were scissored and fixed on a table, then the byssus attachment strength was evaluated using the method developed by Babarro and Comeau (2014) using a portable load meter (DPS-100R; Imada Co. Japan, precision of 0.1N).

### Measurement of shell-closing strength

At the end of experiment, shell-closing strength (SCS) was also assessed on three mussels per tank. SCS is the load force required to open the shell of the mussel by 10 mm with a shell opener. The device for measuring shell-closing strength includes a portable load meter (DPS-100R; Imada Co. Japan, precision of 0.1N) and a shell opener (Nishii Co., Japan). To close the valves, the mussel was soaked in experimental water for 10 min before shell-closing strength measurement. Later, the shell opener was inserted into the crack between the valves, and the load force was determined.

### Statistical analysis

Data were pooled in each tank, and individual tanks of each treatment were considered as replicates (i.e., *N* = 3). Two-way ANOVAs were conducted to evaluate if DO, pH and their interactions affected byssus performance and SCS. When the analysis showed significant interactions, a one-way ANOVA was carried out for evaluating pH effects at each fixed DO level, followed by a Tukey's posteriori HSD test; student *t*-test was applied to test the difference between two DO groups at each fixed pH level, respectively. Three-way ANOVAs were used to evaluate whether time, DO, pH and their interactions affected genes expression. When interactions between the factors was found, the effect of each factor was analyzed independently at each level of the other factors by one-way ANOVA followed by Tukey's posteriori HSD test or student t test and differences among treatments were considered significant when p was less than 0.05. The above analyses were carried out using SPSS18.0, and the values of all parameters are expressed as the means ± S.D. Finally, Principal Component Analysis (PCA) was conducted by XLSTAT® 2014, and a biplot was graphed by integrating both the observations and the measured variables.

## Results

**Figure 1:**
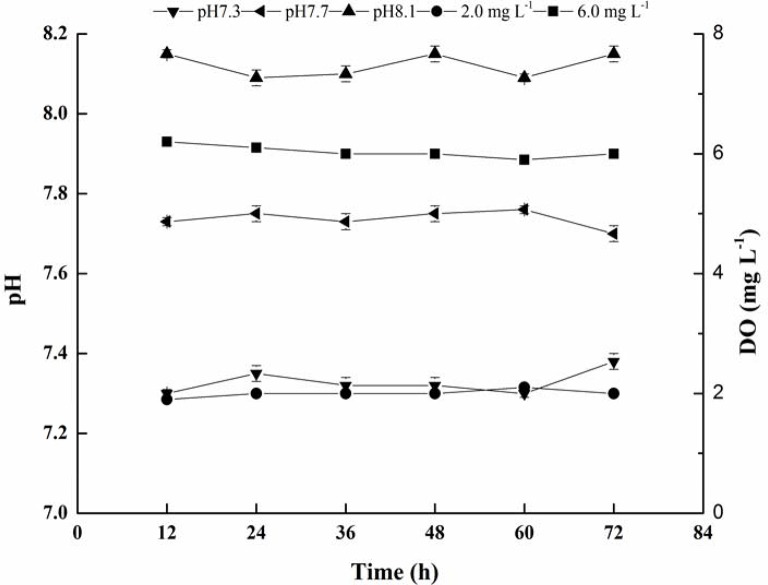
**Mean values of seawater pH and DO during the 72 h of exposure**.

Seawater parameters (DO and pH) targets were achieved in all replicates. During the experiment, salinity was consistently kept at ca. 25‰, and DO concentrations were consistently kept at either 2 mg L^−1^ or 6 mg L^−1^ in the hypoxic and normoxic treatments, respectively., Total alkalinity was maintained at ca. 2,300 μmol kg^−1^. The carbonate chemistry was also maintained at the desired levels (Table 2 and Figure 1).

**Table 2:** **Parameters of seawater carbonate chemistry during the experimental period (***n*** = 3)**.

Treatments pH*DO	**DO (mg L^−1^)**	**Temperature (°C)**	**Salinity (psu)**	**pH_NBS_**	**A_T_ (μmol Kg^−1^)**	**DIC (μmol Kg^−1^)**	**pCO_2_ (μatm)**	**Ωca**	**Ωar**
8.1*2.0	2.01 ± 0.14	20.0 ± 0.2	24.95 ± 0.2	8.11 ± 0.01	2299 ± 21	2072 ± 13	362 ± 11	4.47 ± 0.10	2.81 ± 0.06
7.7*2.0	2.03 ± 0.16	20.1 ± 0.3	25.1 ± 0.2	7.73 ± 0.02	2311 ± 26	2228 ± 15	1005 ± 20	2.02 ± 0.04	1.27 ± 0.03
7.3*2.0	2.04 ± 0.13	20.2 ± 0.1	25.2 ± 0.4	7.32 ± 0.01	2317 ± 28	2351 ± 25	2704 ± 176	0.83 ± 0.03	0.52 ± 0.02
8.1*6.0	6.02 ± 0.15	20.1 ± 0.3	25.0 ± 0.2	8.11 ± 0.01	2304 ± 4	2073 ± 14	356 ± 9	4.54 ± 0.10	2.86 ± 0.06
7.7*6.0	6.03 ± 0.16	20.2 ± 0.2	25.3 ± 0.2	7.71 ± 0.01	2312 ± 26	2224 ± 15	973 ± 34	2.08 ± 0.06	1.31 ± 0.03
7.3*6.0	6.01 ± 0.15	20.1 ± 0.1	25.1 ± 0.3	7.27 ± 0.02	2325 ± 10	2348 ± 27	2681 ± 212	0.84 ± 0.05	0.53 ± 0.03

*DO (mg L^−1^), temperature (°C), salinity, pH_NBS_ and total alkalinity (A_T_, μmol kg^−1^) were used to calculate dissolved inorganic carbon (DIC, μmol kg^−1^), CO_2_ partial pressure (pCO_2_; μatm) and saturation states of aragonite (Ωar) and calcite (Ωca)*.

**Figure 2:**
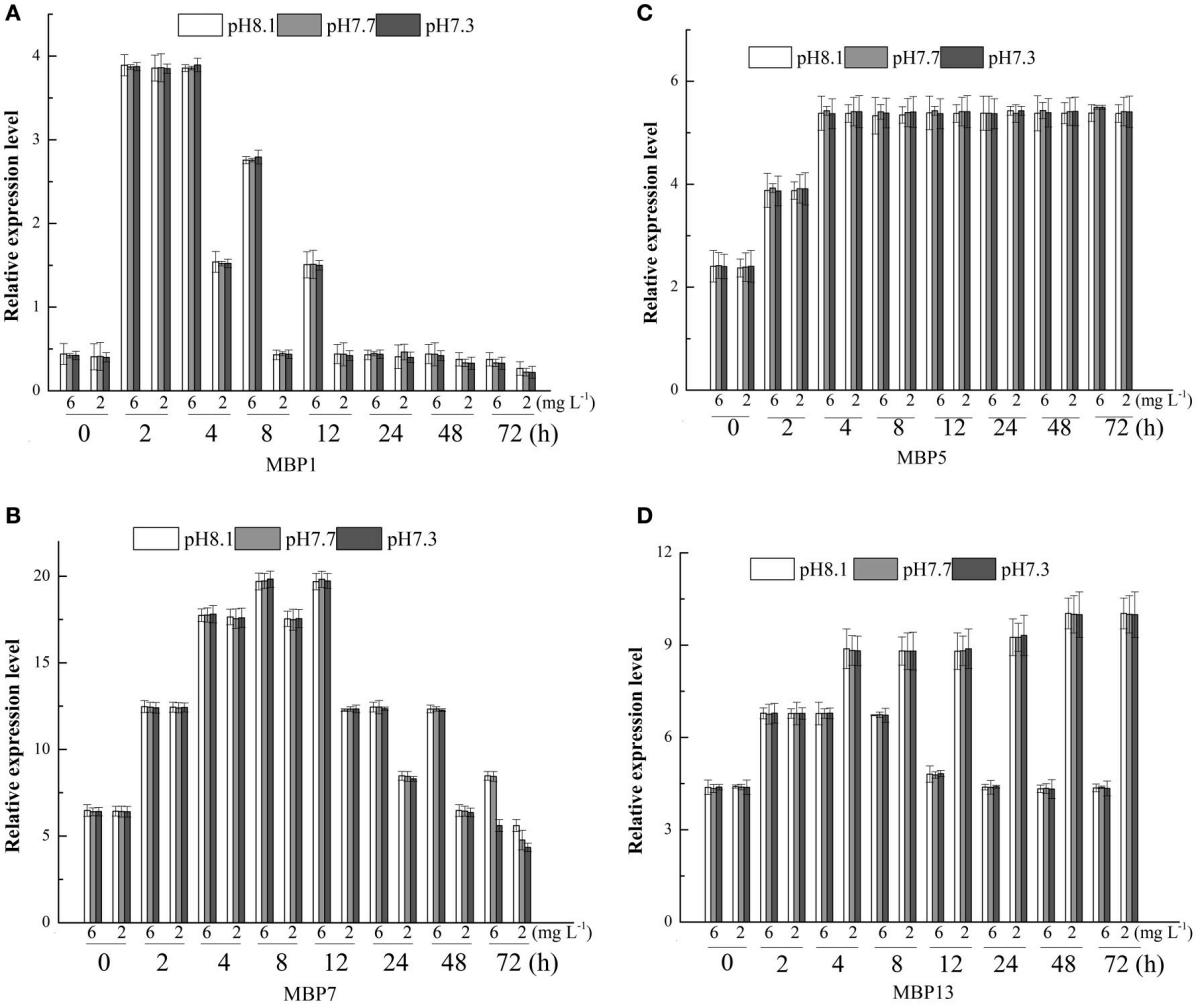
**Real-time PCR analysis of expression of MBP1, 5, 7, 13 (A–D)** in response to three different pH levels and two dissolved oxygen concentration for 72 h.

The relative expression level of the gene associated with mussel byssus protein1 (MPB1) was significantly affected by DO, time and their interactions. There was no significant effect of pH on the relative expression level of this gene (Table 3). The relative expression level of MPB1 fluctuated largely during the experimental period and the relative expression level peaked at 2 h in both DO treatments (Figure 2A). During the first 2 h, there were no significant differences between DO treatments. Between 4 and 12 h, the gene expression levels in the DO 6.0 mg L^−1^ group were significantly higher than in the DO 2.0 mg L^−1^ treatments. At 24 h, no significant differences were found between treatments. At 48 h, although there were no significant differences between various pH groups when DO was 6.0 mg L^−1^. At DO 2.0 mg L^−1^, the gene expression level was significantly higher at pH 8.1 as compared to pH 7.7 and 7.3. At 72 h, the gene expression level at pH 8.1 group was significantly higher than pH 7.7 and 7.3 under both DO levels (Figure 2A).

**Table 3:** **Summary of three-way ANOVA results on effects of pH, dissolved oxygen (DO) and time (T) on gene expressions of mussel byssus protein1, 5, 7, and 13 (MBP1, MBP5, MBP7, MBP13)**.

**Sources**	**df**	**MBP1**			**MBP5**			**MBP7**			**MBP13**		
		**MS**	**F**	**P**	**MS**	**F**	**P**	**MS**	**F**	**P**	**MS**	**F**	**P**
pH	2	0.001	0.166	0.848	0.014	0.214	0.807	0.135	1.066	0.348	0.002	0.016	0.984
DO	1	20.250	2438.739	0.000	0.000	0.004	0.948	304.997	2414.889	0.000	332.546	2236.551	0.000
T	7	31.162	3752.935	0.000	22.369	348.518	0.000	415.032	3286.115	0.000	20.740	139.491	0.000
pH*DO	2	0.001	0.145	0.865	0.012	0.185	0.832	0.108	0.852	0.430	0.000	0.001	0.999
pH*T	14	0.001	0.155	1.000	0.002	0.039	1.000	0.090	0.713	0.757	0.002	0.011	1.000
DO*T	7	4.881	587.828	0.000	0.002	0.011	1.000	35.752	283.076	0.000	24.361	163.838	0.000
pH*DO*T	14	0.000	0.052	1.000	0.001	0.010	1.000	0.067	0.533	0.908	0.001	0.008	1.000

The relative expression level of MBP5 was only significantly affected by time (Table 3). The relative expression level of MBP5 increased with time, peaked at 4 h and then maintained this level until the end of experiment (Figure 2B).

DO, time and their interaction showed significant effects on the relative expression level of MBP7 (Table 3). The peak of gene expression in the 6 mg L^−1^ treatment was observed at 8 h while the peak of expression was observed at 4 h in the 2 mg L^−1^ treatment (Figure 2C). From 8 to 48 h, the gene expression level was significantly higher at 6 mg L^−1^ as compared to the 2 mg L^−1^ treatment. At 72 h, the gene expression levels in the 6 mg L^−1^ treatment were significantly higher at pH 8.1 and 7.7 as compared to pH 7.3. At 2 mg L^−1^, the gene expression level significantly decreased with pH reduction (Figure 2C).

The relative expression level of MBP13 was significantly affected by DO, time and their interaction (Table 3). In the 6 mg L^−1^ treatment, the relative expression level of MBP13 increased within 2 h and maintained at a high level until 12 h, then it declined to the initial value at 48 h. In the 2 mg L^−1^ treatment, the relative expression level of MBP13 increased with time until 48 h and kept a high value until the end of the experiment (Figure 2D). During the first 2 h, there were no significant differences between treatments. From 4 to 72 h, the gene expression level in the 2 mg L^−1^ treatment was higher as compared to the 6 mg L^−1^ treatment (Figure 2D).

**Figure 3:**
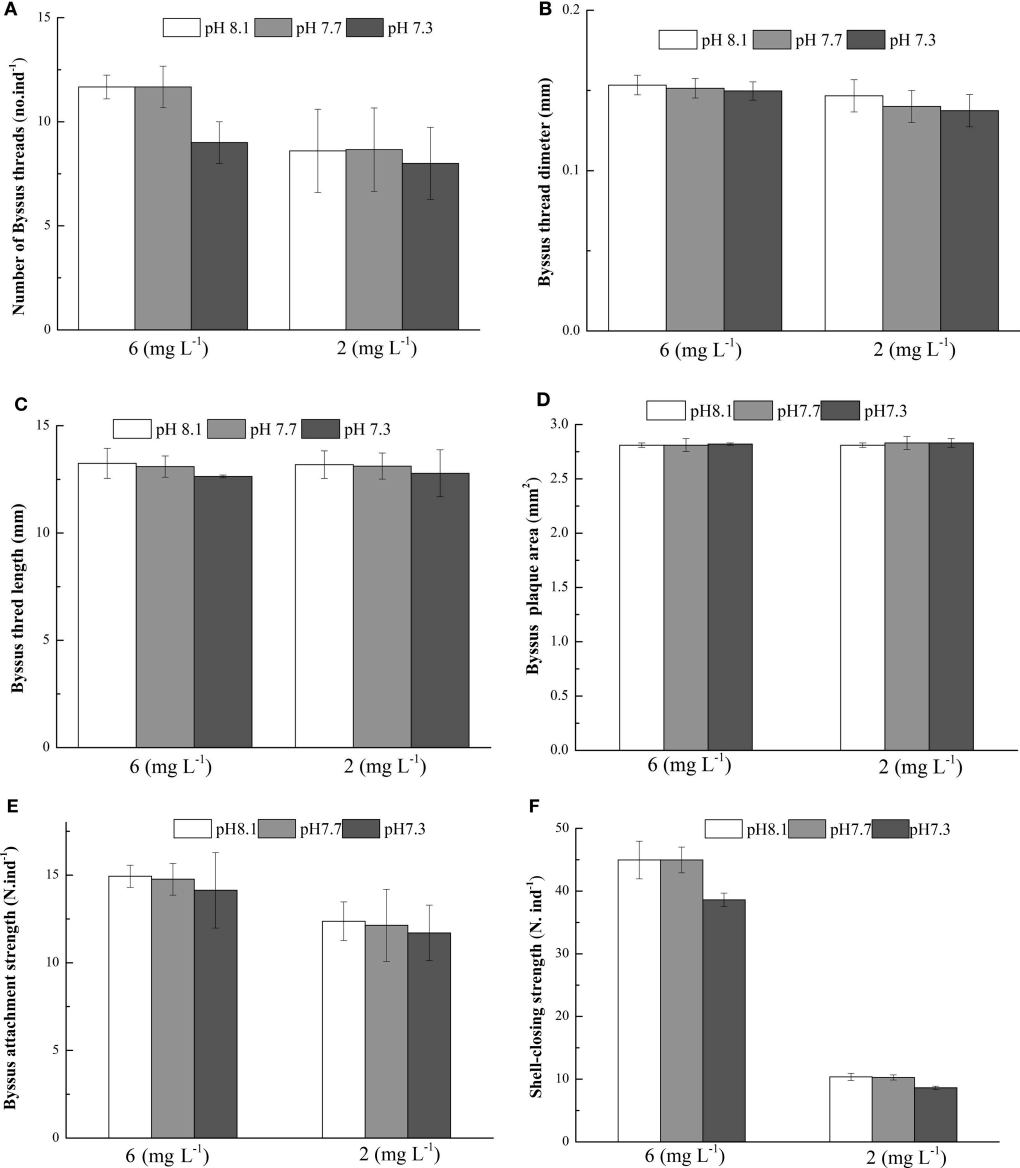
**Byssus thread number (A)**, Byssus threads diameter **(B)**, Byssus thread length **(C)**, Byssus plaque area **(D)**, Byssus attachment strength **(E)** and Shell-closing strength **(F)** of *M. coruscus* exposed to three different pH levels and two dissolved oxygen concentration for 72 h.

At the end of the experiment, significant effects of DO on the number of byssus threads, byssus threads diameter, byssus attachment strength were observed. There were no significant effects of pH and the interaction between pH and DO on these parameters (Table 4). DO, pH, and their interaction did not show significant effects on byssus thread length and byssus plaque area (Table 4). The SCS was significant affected by pH, DO and their interaction. Under normoxic conditions, SCS were significantly higher in pH 8.1 and 7.7 as compared to pH 7.3.Under hypoxic conditions, there were no significant differences between pH treatments (Table 4, Figure 3).

**Table 4:** **Summary of two-way ANOVA results on effects of pH, and dissolved oxygen (DO) on byssus thread number (BTN), byssus thread diameter (BTD), byssus thread length (BTL), byssus attachment strength (BAS), byssus plaque area (BPA) and shell-closing strength (SCS) at the end of the experiment**.

**Sources**	**df**	**BTN**			**BTD**			**BTL**			**BAS**			**BPA**			**SCS**		
		**MS**	**F**	**P**	**MS**	**F**	**P**	**MS**	**F**	**P**	**MS**	**F**	**P**	**MS**	**F**	**P**	**MS**	**F**	**P**
DO	2	34.72	13.587	0.003	0.000	6.738	0.023	0.006	0.013	0.910	30.42	13.727	0.003	0.001	0.411	0.533	4828.169	1931.697	0.000
pH	1	1.722	0.674	0.528	6.572E-5	0.963	0.410	0.427	0.945	0.416	0.932	0.42	0.666	0.000	0.137	0.873	24.855	9.944	0.003
pH*DO	2	0.056	0.022	0.979	1.372E-5	0.201	0.821	0.016	0.036	0.965	0.052	0.023	0.977	6.667E-5	0.034	0.966	16.154	6.463	0.012

**Figure 4:**
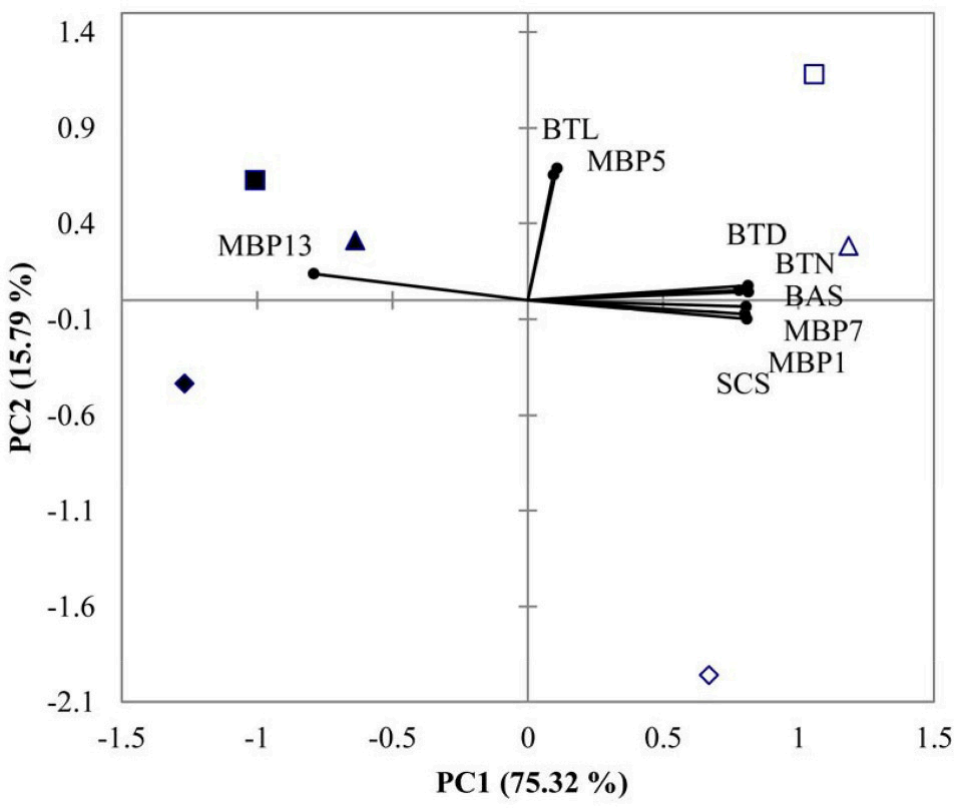
**PCA results of ***M. coruscus*** exposed to three different pH levels and two dissolved oxygen concentration at 72 h**. (▴2 mg L^−1^ × pH 8.1, Δ6 mg L^−1^ × pH 8.1, ■2 mg L^−1^ × pH 7.7, □6 mg L^−1^ × pH 7.7, ♦2 mg L^−1^×pH 7.3, ♢6 mg L^−1^×pH 7.3). Both the loadings of the variables (•) and the scores of the experimental conditions were shown. BTN, byssus thread number, BTD, byssus thread diameter, BTL, byssus thread length, BAS, byssus attachment strength, SCS, shell-closing strength and MBP, mussel byssus protein.

PCA showed that 91.11% of total variance was explained by the two principal components (Figure 4). PC1 accounted for 75.32% of the total variance, and the most distinct response referred to the separation between the two DO treatments. PC2 explained 15.79% of the total variance, and separated low pH 7.3 from the two other pHs (Figure 4).

## Discussion

*M. coruscus* could not only survive but also secret byssus under short-term hypoxia and low pH exposure. This suggests that this species is partly adapted to such short term fluctuations in pH and DO (Gobler et al., 2014). However, the shell-closing strength was weakened upon exposure to these conditions, showing that short term exposure to low pH and DO can impair the mussel's defense capacity.

The present study examined the changes of relative gene expressions of MBP1, MBP5, MBP7, and MBP13 associated with byssus secretion in the mussel *M. coruscus* under low pH and DO conditions. Among them, both MBP1 and MBP5 proteins have a collagen domain-containing protein (Qin et al., 2016). Sun et al. (2014) speculated these collagen domain-containing proteins might be crucial for the tenacity of byssus and probably participated in the cross-linking to mediate the interaction between the threads and plaque. The present study showed that DO was the driving factor influencing levels of mRNA transcript for MBP1&7, whereas low pH aggravated the sensitivity of *M. coruscus* to hypoxia partly. In our previous studies, hypoxia and low pH reduced the metabolic rate of *M. coruscus* (Wang et al., 2015; Sui et al., 2016a). Thus, reductions in mussel byssus protein expressions (i.e., MBP1&7) under hypoxic conditions may be due to metabolic depression, and OA may aggravate such depression. Thomsen and Melzner (2010) observed increased energy expenditures and decreased O:N ratios of *M. edulis* under increasing pCO_2_ conditions, indicating enhanced protein metabolisms. Thus, more energy may be allocated to intracellular pH regulation and protein synthesis may be reduced, resulting in a lower byssus production. MBP1 is only detected in plaque (Sun et al., 2014). As a consequence, the downregulation of the expression implied the seawater acidification and hypoxic may lead to a weakening of the formation of mussel plaque. The relative expression level of MBP5 was significantly affected by time only. This highlights the importance to consider multiple genes when studying the impact of seawater acidification and hypoxia. MBP7 is only identified in thread (Sun et al., 2014). This protein has a von Willebrand factor type A domains (VWA domains, Sun et al., 2014). The VWA domains is made of approximately 200 amino acid residues and is usually encountered in extracellular proteins (containing collagens, integrin, matrilin, complement factors) and mediated the adhesion via metal ion-dependent adhesion sites (Bork, 1991; Colombatti et al., 1993; Perkins et al., 1994). Accordingly, Sun et al. (2014) hypothesized that MBP7 would be a collagen-like, VWA domaining protein and could played an important role in the adhesion of byssus. In the present study, expression of MBP7 was significantly reduced after 8 h exposure to hypoxic conditions. This suggests that an exposure longer 8 h to hypoxia may have adverse effect on byssus thread formation. MBP13 was identified in byssus plaque. It encodes a 181-aa-long protein precursor, including a 17-aa-long signal peptide (Sun et al., 2014). There is a Cu/Zn superoxide dismutase-like domain in the mature peptide of MBP13, which implied MBP13 could protect damages from reactive oxygen species by catalyzing the dismutation of superoxide into molecular oxygen and hydrogen peroxide (Schininà et al., 1985, 1989; Parge et al., 1992). Mussel adhesion depends on the process of DOPA oxidation and subsequent dopaquinone formation (Anderson et al., 2010). DOPA-rich byssus protein is mainly maintained in a reducing state before solidification outside substratum (Yu et al., 2011). The presences of superoxide dismutase domain in MBP13 could play a key role in redox balance to prevent premature oxidation of DOPA-rich protein (Sun et al., 2014). We found that the expression of MBP13 gene in hypoxic treatment was significantly higher than that in normoxic treatment after 4 h of exposure. This could suggest that mussels exposed to hypoxia need more MBP13 to eliminate the oxygen-mediated free radicals. This is supported by our previous observation that *M. coruscus* produced more oxygen-mediated free radicals when exposed to seawater acidification and hypoxia (Sui et al., 2016b).

Byssus production in mussels can be affected by many factors including temperature, pH (Li et al., 2015), salinity (Wang et al., 2012) and current (Taylor et al., 1997). In our experiment, we showed that hypoxia weaken mussel byssus production. Under hypoxic conditions, byssus thread number, diameter, plaque area were significantly reduced. Similar results were reported in the green mussel *P. viridis* by Wang et al. (2012) who demonstrated that hypoxia resulted in a reduced number of byssus threads and thinner diameter. Similar results were also reported for the mussels *Dreissena polymorpha* (Clarke and McMahon, 1996) and *M. edulis* (Reish and Ayers, 1968). In our study, acidification had no effect on these parameters. This is only in partial agreement with the work on the pearl oysters *P. fucata* held at pH 7.8 and pH 7.6. In this study, pH had no effect on the number of byssus threads, but byssus produced by that oysters were significantly thinner at 7.6 as compared to other pH treatments (Welladsen et al., 2011). Assuming that byssus production mirrors mussel adaptive capacity, it would mean that *M. coruscus* is less sensitive to pH changes than *P. fucata*.

Previous studies on bivalves showed that the high tensile strengths of byssus prevent them from being removed by wave action or predators (Steffani and Branch, 2003; Moeser et al., 2006; Dolmer and Svane, 2012). Seguin-Heine et al. (2014) reported that mussels maintained in more turbulent areas can produce more and stronger byssus compared to those in more sheltered environments. In the present experiment, hypoxia brought adverse effects on byssus attachment strength suggesting that hypoxia could weaken mussel defense capability.

Shell-closing strength is an important physiological indicator as healthy mussels generally keep their valves tightly closed in reaction to predators and waves (Aoki et al., 2010). Therefore, the shell-closing strength in mussels can mirror its defense performance. We showed that shell-closing strength was significantly affected by DO, pH and their interaction. This suggests that seawater hypoxia and acidification has the potential to impair mussel defense capacity. More work is needed to better understand the interactive effect of the two drivers.

PCA allowed to discriminate normoxic treatments from hypoxic treatments by PC1. Normoxic treatments were associated with higher values of BTD, BTN, BAS, MPB7, MPB1, and SCS whereas hypoxia was associated with high MPB13. PC2 separated low pH (7.3) from higher pH (7.7 and 8.1) treatments (Figure 4). By integrating ANOVA and PCA results, the characteristics of the defense response to hypoxic exposure were lower BTD, BTN, BAS, MPB7, MPB1, and SCS associated with higher MPB1.

It is well documented that seawater acidification and hypoxia has the potential to harm many marine species (e.g., Wu, 2002; Orr et al., 2005; Diaz and Rosenberg, 2008; Keppel et al., 2015; Wit et al., 2016). However, the interaction between these two drivers has received limited attention. This study stressed the importance of evaluating the combined effects of hypoxia and acidification on the defense responses in mussels. We showed that some aspects of the defense responses of *M. coruscus* was impaired by decreasing DO and pH. Depressed byssus attachment strength and shell-closing strength may enable predators and water current to remove mussels from the substrates more easily. Hence, *M. coruscus* would be more exposed to their predators under hypoxia and acidification in estuarine and coastal waters.

## Author contributions

YW Designed and led the study, YS, YL, XZ, MH, and FW performed the experiments, YS, YL, XH, SD, JL, WL, and YW analyzed data and wrote the manuscript.

### Conflict of interest statement

The authors declare that the research was conducted in the absence of any commercial or financial relationships that could be construed as a potential conflict of interest. The reviewer YT and handling Editor declared their shared affiliation, and the handling Editor states that the process nevertheless met the standards of a fair and objective review.
